# VISION LOSS AND PSYCHOSOCIAL FUNCTIONING: WHAT’S THE CONNECTION?

**DOI:** 10.1093/geroni/igad104.0528

**Published:** 2023-12-21

**Authors:** Louay Almidani, Rhonda Miller, Varshini Varadaraj, Aleksandra Mihailovic, Pradeep Ramulu

**Affiliations:** Wilmer Eye Institute, Baltimore, Maryland, United States; Wilmer Eye Institute, Baltimore, Maryland, United States; Johns Hopkins University, Baltimore, Maryland, United States; Wilmer Eye Institute, Baltimore, Maryland, United States; Wilmer Eye Institute, Baltimore, Maryland, United States

## Abstract

This study assessed the associations between vision impairment (VI) and psychological and social function in older adults. We conducted a cross-sectional analysis of the National Health and Aging Trends Study, a nationally representative sample of Medicare beneficiaries aged 65 years and older. VI was measured using categorical and continuous scales. Depression and anxiety symptoms were assessed via the Patient Health Questionnaire for Depression and Anxiety (PHQ-4) questionnaire. Social isolation and severe social isolation were defined based on responses to questions on living arrangement, frequency of communication with others, and participation in activities. Results showed that depressive symptoms, social isolation, and severe social isolation were more prevalent in adults with any objective VI measure compared to those without impairment. In adjusted models, objective vision measures were not associated with higher odds of depression or anxiety symptoms, though both outcomes were strongly associated with self-reported VI (OR: 1.82 [95% CI: 1.27 – 2.60], OR: 1.81 [95% CI: 1.30 – 2.53], respectively). In contrast, worse distance and near acuity (per 0.1 logMAR), and worse contrast sensitivity (per 0.1 logCS units) were associated with higher risk of being severely socially isolated (RRR: 3.79 [95% CI: 1.13 – 12.78], 3.26 [95% CI: 1.16 - 9.17], 2.82 [95% CI: 1.11 – 7.18], respectively), however, no associations were found with self-reported VI. This study demonstrates that worse vision is associated with symptoms of depression, anxiety, and social isolation among older US adults. Approaches are needed to enhance the psychological and social well-being of individuals with VI.

